# Modified PCR-based assay for the differentiation of members of *Anopheles fluviatilis* complex in consequence of the discovery of a new cryptic species (species V)

**DOI:** 10.1186/s12936-020-03172-1

**Published:** 2020-02-27

**Authors:** Om P. Singh, Nutan Nanda, Dinesh Chandra, Deepa Jha, Tridibes Adak, Virendra K. Dua, Sarala K. Subbarao

**Affiliations:** 1 National Institute of Malaria Research, Sector 8, Dwarka, Delhi 110077 India; 2 National Institute of Malaria Research, Field Station, BHEL Complex, Sector-III Dispensary, Ranipur, Hardwar, 249403 India; 3 Indian Council of Medical Research, V. Ramalinaswami Bhawan, Ansari Nagar, New Delhi, 110029 India

**Keywords:** *Anopheles fluviatilis*, Species complex, Sibling species, ASPCR

## Abstract

**Background:**

*Anopheles fluviatilis* is a species-complex comprising of four cryptic species provisionally designated as species S, T, U and V. Earlier, a 28S-rDNA based allele-specific polymerase chain reaction (ASPCR) assay was developed for the differentiation of the then known three members of the *An. fluviatilis* complex, i.e., species S, T, and U. This assay was modified in consequence of the discovery of a new cryptic member, species V, in the Fluviatilis Complex to include identification of new species.

**Methods:**

In the modified procedure, the ASPCR assay was performed first, followed by restriction digestion of PCR product with an enzyme *Bam*H I, which cleaves specifically PCR amplicon of species V and the resultant PCR–RFLP products can differentiate all the four cryptic members of the complex. Morphologically identified *An. fluviatilis* samples were subjected to sibling species identification by modified PCR-based assay and standard cytotaxonomy. The result of PCR-based assay was validated through cytotaxonomy as well as DNA sequencing of some representative samples.

**Results:**

The modified PCR-based assay differentiates all four sibling species. The result of modified PCR-based assay tested on field samples was in agreement with results of cytotaxonomy as well as DNA sequencing of representative samples.

**Conclusions:**

The modified PCR-based assay unambiguously differentiates all four known members of the *An. fluviatilis* species complex. This assay will be useful in studies related to bionomics of members of the Fluviatilis Complex in their role in malaria transmission.

## Background

*Anopheles fluviatilis* is one of the most important vectors of malaria in India. Earlier, this species was recognized as a complex of three cryptic species, species S, T and U, by Subbarao et al. [1] which varied in biological characteristics that are known to have epidemiological implication in malaria transmission; mainly, host-preference and their vectorial potential [2, 3]. Species S is highly anthropophagic and is a highly efficient malaria vector [2, 4, 5], whereas species T and U are almost zoophilic, and considered as poor vectors [4, 6, 7]. Subsequently, in the year 2013, a new cryptic species was reported in this complex which has been designated as species V [8]. This new species is characterized by having two fixed paracentric inversions s1 and S present on arms 2 and 3, respectively of polytene chromosome complement, and also by fixed nucleotide differences in the D3 domain of 28S- and ITS2-rDNA [8]. The biology of this new species and its role in malaria transmission is not investigated so far, except that in Hardwar where the new species was identified, species V had 0.04 human blood index while for species T and U collected from the same area it was 0.0 [8].

Identification of members of the Fluviatilis Complex is crucial in vector control programme owing to contrasting differences in the biological characteristics that are epidemiologically important. Prior to the discovery of new cryptic species (species V) in the Fluviatilis Complex, an allele-specific PCR assay (ASPCR) was developed by Singh et al. [9] for the differentiation of the then known three members of the complex, i.e., species S, T, and U, which was based on species-specific differences in nucleotide sequences in D3 domain of 28S ribosomal DNA (rDNA). The discovery of additional cryptic species (species V) in the Fluviatilis Complex [8] has complicated further studies on the bionomics of *An. fluviatilis* in the absence of a PCR-based assay for the differentiation of all four members of the Fluviatilis Complex. The only available diagnostic tool for the identification of species V is cytotaxonomy which can be applied only on half-gravid female mosquitoes (Christophers’ stage III). Additionally, this method is tedious and requires a highly skilled person to read the banding pattern on the polytene chromosome. Therefore, the existing PCR-based assay was modified to discriminate all the four members of the Fluviatilis Complex. Moreover, unlike cytotaxonomy, this assay can be applied to all stages and both sexes of dead or alive mosquitoes.

## Methods

### Study sites and sample collection

Two *An. fluviatilis* sensu lato (s.l.) populations, one from district Hardwar, Uttarakhand (30.0° N, 78.2° E), where species T, U, and V are prevalent [8] and the other from district Sundergarh, Odisha (22.1240° N, 84.0432° E), where species S is prevalent in sympatric association with T [1, 5], were selected for this study. *Anopheles fluviatilis* s.l. was collected from villages Dargahpur, Auspur and Ismailpur of district Hardwar, from where species V is reported to exist and found in sympatric association with species T and U. Mosquitoes were collected between 6:00 to 8:00 A.M. from cattle sheds and human dwelling using an aspirator and torchlight.

### Sample processing and cytotaxonomy

Ovaries were extracted from half-gravid (Christophers’ stage III) mosquitoes. Fully-fed mosquitoes with under-developed ovarian stage were allowed to attain half-gravid condition at room temperature before extraction of ovaries. The procedure of processing of half-gravid mosquitoes for cytotaxonomy was as described by Subbarao et al. [1]. The mosquitoes which were not in an appropriate ovarian developmental stage suitable for cytotaxonomy were kept in a cage, transported to laboratory and allowed to lay their eggs. These mosquitoes were later fed on the rabbit and kept in an insectary maintained at temperature 25 ± 1 °C and relative humidity of 70 ± 5% for ovarian development. After the attainment of ovarian development until the Christophers’ stage III, ovaries were removed from individual mosquitoes and fixed in modified Carnoy’s fixative (3:1 ethanol: glacial acetic acid). The remaining carcasses of individual mosquitoes were preserved in isopropanol and later used for DNA isolation. Ovaries from individual mosquitoes were used for the preparation of nurse cell polytene chromosomes according to the method of Green and Hunt [10]. Polytene chromosomes were examined in Zeiss Axioplan Universal microscope for the genotyping of q1, r1, s1 and S inversions following Nanda et al. [8].

Mosquitoes from which ovaries could not be extracted were preserved in isopropanol *in toto* for DNA isolation. All the mosquitoes were identified morphologically following Christophers’ key [11], prior to ovary extraction or before preserving them in isopropanol for DNA isolation.

### DNA isolation

DNA from individual mosquitoes was isolated using the method described by Livak [12].

### Development of PCR-based molecular diagnostics

For the development of PCR-based strategy for the identification of all members of the Fluviatilis Complex, DNA sequences of D3 domain of 28S-rDNA of species S, T, U and V of the Complex [8, 9] were aligned (GenBank accession numbers AF437880, AF437881, AF437882 and JF327858, respectively). The species-specific differences in nucleotide sequences among members of the complex are shown in Table 1. The nucleotide differences which are specific to species V are at base position numbers 60, 62, 94 and 136 (Table 1). These SNPs were either not found suitable for designing primers that can produce species V specific amplicon, easily distinguishable from other species-specific amplicons on normal agarose gel, or primers designed produced excessive primer dimers and suppression of other species-specific amplicons. Therefore, the sequences were analysed for the presence of unique restriction sites present in species V, which can be used for discrimination of new species. Analysis of DNA sequences of all members of the Fluviatilis Complex using an online tool available at http://insilico.ehu.es/restriction/compare_seq/ revealed the presence of two unique restriction sites *Bam*H I (5′-G↓GATCC-3′) and XhoII (5′-R↓GATCY-3′) in species V which were absent in rest all other three cryptic species. Of the two enzymes, *Bam*H I was selected for RFLP assay for two reasons: for not having ambiguous bases in recognition site and considering the cost concern. The *Bam*H I is amongst the cheapest available restriction enzymes (US$ 0.005 per unit, New England Biolabs). The expected size of fragments after digestion of 375 bp ASPCR product with *Bam*H I, in case of species V, is 155 ± 2 and 220 ± 2 bp. These fragments are readily distinguishable from species S and T specific bands, 128 bp and 295 bp, respectively, in existing ASPCR.

**Table 1:** The variable nucleotide bases in D3 domain of 28S-rDNA in members of *Anopheles fluviatilis* complex

	Base position number						
Species	0	0	0	0	0	0	1
	6	6	7	7	9	9	3
	0	2	6	7	2	4	6
Species S	C	G	A	C	T	G	C
Species T	–	–	G	A	G	–	–
Species U	–	–	G	A	A	–	–
Species V	A	C	G	A	–	A	T

Base position numbers are in respect to GenBank accession numbers AF437880 (species S), AF437881 (species T), AF437882 (species U) and JF327858 (species V)

### Molecular identification of cryptic species

DNA of all samples were subjected to modified PCR based assay for the identification of cryptic species. In the modified procedure, an initial PCR assay was carried out using the original species-diagnostic ASPCR assay described by Singh et al. [9]. Briefly, the PCR reaction mixture (25 μl) contained 1.5 mM of MgCl_2_, 200 μM of each dNTP and 0.625 units of hotstart taq polymerase (AmpliTaq Gold). Primers used were: 1.5 μM of D3A (forward, 5′-GAC CCG TCT TGA AAC ACG GA-3′), 1.5 μM of D3B (reverse, 5′-TCG GAA GGA ACC AGC TAC TA-3′), 1.5 μM of AFS (forward, 5′-CTG GAA ACC CAC AGG CAC-3′), and 1.4 μM of AFT (reverse, 5′-TAC CCG TAA TCC CGC AC-3′). PCR conditions were initial denaturation at 95 °C for 5 min, 35 cycles each at 95C for 30 s, 55 °C for 30 s and 72 °C for 60 s, and a final extension at 72 °C for 7 min. D3A and D3B are universal primers that produce 375 bp amplicon, while AFS and AFT are species-specific primers for species S and T which produce 295 bp (with D3B) and 128 bp amplicons (with D3A), respectively. Subsequently, 15 μl of the amplified ASPCR product was digested with 5 units of BamHI-HF (New England Biolabs Inc.) for 2 h at 37 °C followed by denaturation of the enzyme at 80 °C for 10 min. The resultant ASPCR-RFLP products were electrophoresed on 2.0% agarose gel containing ethidium bromide and visualized under ultraviolet rays in the gel documentation system.

### DNA sequencing

Representative samples of each sibling species as identified by PCR based method were sequenced for the D3 domain of 28S rDNA for molecular validation of the assay. Sequencing was performed following the method of Singh et al. [13].

## Results

**Fig. 1:**
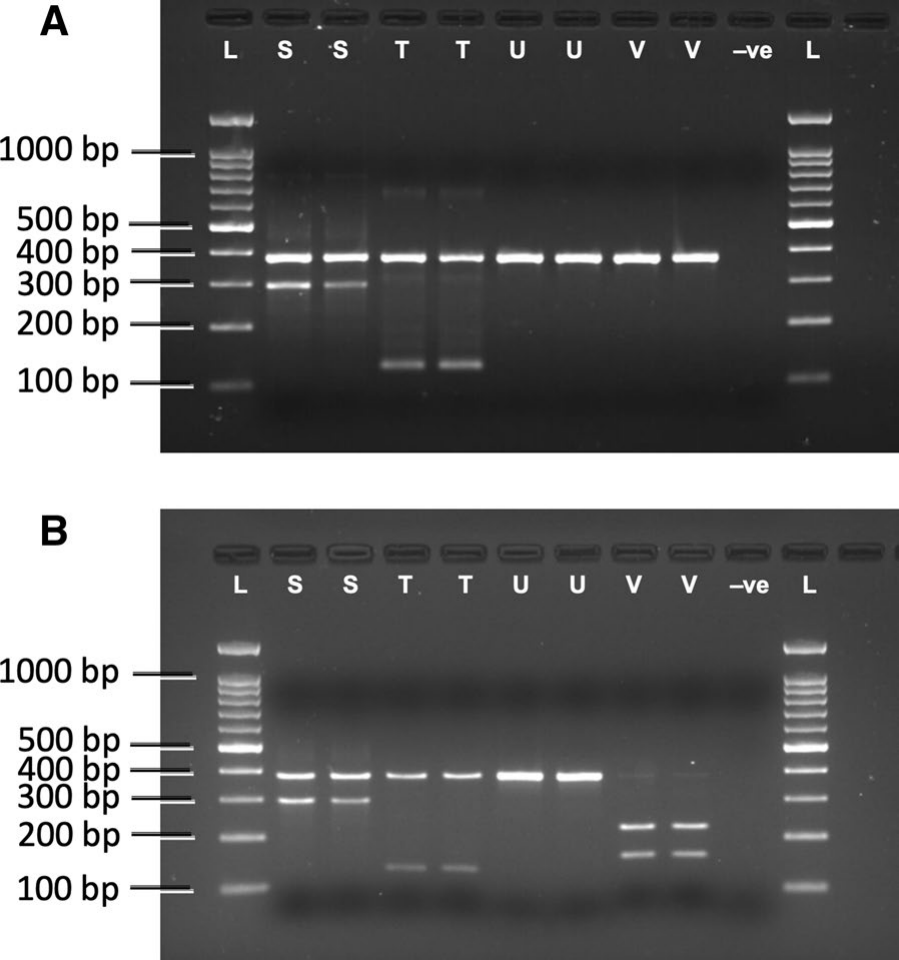
PCR-based species diagnostic assay for the differentiation of members of *An. fluviatilis* complex. **a** Result of ASPCR [1] without restriction digestion. It may be noted that the distinction of species U and V is not possible with this ASPCR. **b** Banding pattern of ASPCR products after *Bam*H I-digestion. The *Bam*H I cleaves PCR amplicon of species V only and can be differentiated from other species (*L* 100 bp DNA ladder, *S* species S, *T* species T, *U* species U, *V* species V; *−ve* negative control, without DNA. It may be noted that *Bam*H I cleaves only species V)

The gel photographs of ASPCR products before and after digestion with *Bam*H I are shown in Fig. 1a, b, respectively. Figure 1a shows that species U and V both has 375 bp and cannot be differentiated by ASPCR alone but when digested with restriction enzyme *Bam*H I the ASPCR derived amplicon is cleaved into fragments of 155 and 220 bp only in species V (Fig. 1b), as expected from sequenced data. However, it does not affect ASPCR derived amplicons of other species. Thus, the modified PCR based assay unambiguously differentiates all four sibling species where the criteria for the assignment of different species were: 295 + 375 bp for species S, 128 + 375 bp for species T, 375 bp only for species U and 155 + 220 bp for species V.

A total of 409 samples of morphologically identified *An. fluviatilis* were subjected to modified PCR assay for sibling species identification. Out of 387 samples collected from Hardwar, 74 were identified as species T, 224 as species U and 89 as species V. All 22 DNA samples from Sundergarh (state Odisha) were identified as species S. Among all mosquitoes, ovaries from only 120 were examined for polytene chromosome. The association of chromosomal inversion genotypes and the result of diagnostic PCR assay are presented in Table 2, which shows that the results of PCR based assay were in agreement with the results of cytotaxonomic identification.

**Table 2:** Results of modified PCR-based assay and cytotaxonomy, and their association

Locality	Species identified by molecular assay	Chromosomal inversion genotypes (species)					Total
		2q^1^ + r^1^ + s^1^; 3 + S (T)	2 + q^1^r^1^ + s^1^; 3 + S (U)	2 + q^1^ + r^1^s^1^; 3S (V)	2 + q^1^ + r^1^ + s^1^; 3 + S (S)	Not identified	
Hardwar, Uttarakhand)	T	21	–	–	–	53	74
	U	–	52	–	–	172	224
	V	–	–	25	–	64	89
Sundergarh, Orissa	S	–	–	–	22	–	22
Total		21	52	25	22	289	409

Of the 116 specimens from Hardwar and Haldwani (state Uttarakhand) that were identified as species U by ASPCR in the previous study [9] and could not be identified cytotaxonomically, when subjected to modified PCR-based assay, six specimens were identified as species V. This indicates that species V was prevalent in the previous survey but could not be identified as both species U and V exhibit 375 amplicon in ASPCR assay.

A total of five samples each of species S, T and U and 21 samples of species V were sequenced for the D3 domain of 28S rDNA. The sequencing results were in agreement with the results of PCR-based assay. The DNA sequences have been submitted to GenBank (accession numbers: MT022523–MT022559). The sequences of species S, T, U and V were identical to their respective reference GenBank sequences (AF437880, AF437881, AF437882 and JF327858, respectively).

## Discussion

*Anopheles fluviatilis*, a major vector of malaria in India [14], has now been established as a complex of four cryptic species [8]. As they differ in malaria transmission potential and host preference, identification of these species is crucial for the vector control programme. Previously only three members were recognized in the Fluviatilis Complex, i.e., species S, T & U, based on the fixed species-specific paracentric inversions in arm 2 of the polytene chromosome complement [1]. Subsequently, an ASPCR technique was developed by Singh et al. [9] to differentiate the three known members of morphologically identified *An. fluviatilis* on the basis of species-specific differences in the nucleotide sequence of D3 domain of 28S rDNA, which is an easier and convenient tool as compared to cytotaxonomy. Subsequently, in the year 2013, a new cryptic species (V) was reported in the Fluviatilis Complex [8]. Therefore, the old ASPCR was modified to include identification of this newly discovered species V.

The presence of cryptic species has been very frequently recognized in anophelines. In India, all major malaria vectors, except *Anopheles stephensi,* are known to be complexes of several sibling species [15]. Molecular tools have become most convenient tool for the identification of cryptic species and rDNA has been frequently used for such purpose. Among rDNA units, internal transcribed spacer unit II (ITS2) being highly variable among different species has been frequently used for molecular taxonomy and species diagnostics. In the case of *An. fluviatilis,* first species diagnostic assay was developed by Manonmani et al. [16] based on differences in ITS2 sequences of species S and T, which can differentiate species S from T and U but cannot differentiate T and U. Thereafter, Singh et al. [9] developed a species diagnostic assay based on D3 domain of 28S rDNA which could differentiate all the then known three sibling species (S, T and U). 28S rDNA being comparatively highly conserved, has not been extensively used for molecular identification of sibling species being, where only D1 to D3 domains (out of 7 domains) are variable, However, D3 domain of 28S rDNA have been used successfully for the identification of members of several anophelines, such as *Anopheles minimus* [17], *An. fluviatilis* [9], *Anopheles funestus* [18]*, Anopheles maculatus* [19], *Anopheles nili* [20]*, Anopheles subpictus* [21]. However, this region has not been used for the *Anopheles gambiae* complex, an expensively studied malaria vector.

Conventional morphological taxonomy generally cannot differentiate cryptic species and in some cases, it is problematic even for the differentiation of closely related species [22, 23]. PCR-based assays for the identification of biological species based on rDNA have emerged as an important tool due to the conserved nature of such repetitive DNA in an interbreeding population due to concerted evolution which leads to intraspecific homogenization of repetitive sequence arrays. Members of the Fluviatilis Complex can be recognized either by using cytotaxonomy or PCR-based assay. Cytotaxonomy, which played a crucial role in unravelling sibling species in the majority of malaria vectors, has certain practical limitations as discussed earlier. PCR-based techniques, on the other hand, can identify all life-stages of mosquitoes, dead or alive, and relatively easy to perform. However, precaution should be taken to apply such techniques (cytological as well as molecular techniques) on morphologically correctly identified species, because incorrect morphological identification prior to use of such assays can seriously lead to incorrect classification of species. For example, in a recent study carried out in South Africa, it was found that standard PCR assays developed for the members of *An. gambiae* complex and for *An. funestus* group wrongly classifies three species (*Anopheles rufipes, Anopheles rhodesiensis* and *Anopheles pretoriensis*) belonging to *An. funestus* group and four species (*Anopheles squamosus An. pretoriensis, Anopheles listeri* and *An. rufipes*) as members of *An. gambiae* complex, when tested on a total of 11 morphological species [23]. Similarly, standard cytotaxonomy for the *An. fluviatilis* complex wrongly classifies morphologically misidentified *An. minimus* s.l. as *An. fluviatilis* U [13]. Therefore, this PCR diagnostic technique should be used on correctly identified morphological species as *An. fluviatilis*.

## Conclusion

In consequence of the addition of a new cryptic species (species V) in the Fluviatilis Complex, existing ASPCR method developed by Singh et al. [9], for the differentiation of the then known three members of the species complex, was modified to include identification of species V. The modified PCR-based assay unambiguously differentiates all four known members of the *An. fluviatilis* species complex. This assay will be useful in studies related to bionomics of members of the Fluviatilis Complex in their role in malaria transmission.

## Data Availability

All data generated or analysed during this study are included in this published article.

## Abbreviations

ASPCR: Allele-specific polymerase chain reaction
bp: Base pair
dNTP: Deoxyribonucleotide triphosphate
ITS2: Internal transcribed spacer 2
PCR: Polymerase chain reaction
rDNA: Ribosomal DNA
RFLP: Restriction fragment length polymorphism
